# Recovery of regional myocardial function and myocardial oedema following reperfused acute myocardial infarction

**DOI:** 10.1186/1532-429X-14-S1-O64

**Published:** 2012-02-01

**Authors:** Ananth Kidambi, Adam N Mather, Peter Swoboda, Manish Motwani, Timothy Fairbairn, John P Greenwood, Sven Plein

**Affiliations:** 1 Multidisciplinary Cardiovascular Research Centre & Leeds Institute of Genetics, Health and Therapeutics, University of Leeds, Leeds, UK; 2 Hull & East Yorkshire Cardiothoracic Centre, Castle Hill Hospital, Kingston-upon-Hull, UK

## Summary

We investigated the relationship between myocardial oedema and recovery of regional myocardial function in patients with acute reperfused myocardial infarction (MI).

Early after acute MI, oedema in the peri-infarct zone, as delineated by T2 weighted (T2w) cardiovascular magnetic resonance (CMR) imaging, demonstrated significantly reduced strain as compared to remote myocardium. The recovery of regional function in the peri-infarct zone closely followed the resolution of hyperenhancement on T2w CMR. In addition, both transmural and subendocardial infarcts showed a degree of functional recovery after acute MI.

## Background

Myocardial tissue oedema is a feature of acute reperfused myocardial infarction, and contributes to stunning of viable peri-infarct myocardium (the ‘area at risk’). Regression of oedema on T2w CMR imaging is related to improved myocardial contractility post MI in animal models. Whether a similar relationship between myocardial oedema and regional myocardial function exists in man is not currently known. We hypothesised that the resolution of tissue oedema correlates with recovery of regional contractile function.

## Methods

We studied patients after primary percutaneous coronary intervention for first ST-elevation MI. Patients underwent CMR with T2w, myocardial tagging and late gadolinium enhancement imaging at median 2, 30 and 90 days post reperfusion. Infarct size, regional circumferential strain, intensity and volume of myocardial oedema were measured for infarct zone, peri-infarct zone (area at risk) and remote myocardium. Oedema and infarction were defined as zones with signal intensity 2 standard deviations above remote myocardium in T2w and LGE imaging respectively. T2w signal intensity was corrected using a ratio to remote myocardium.

## Results

**Figure 1:**
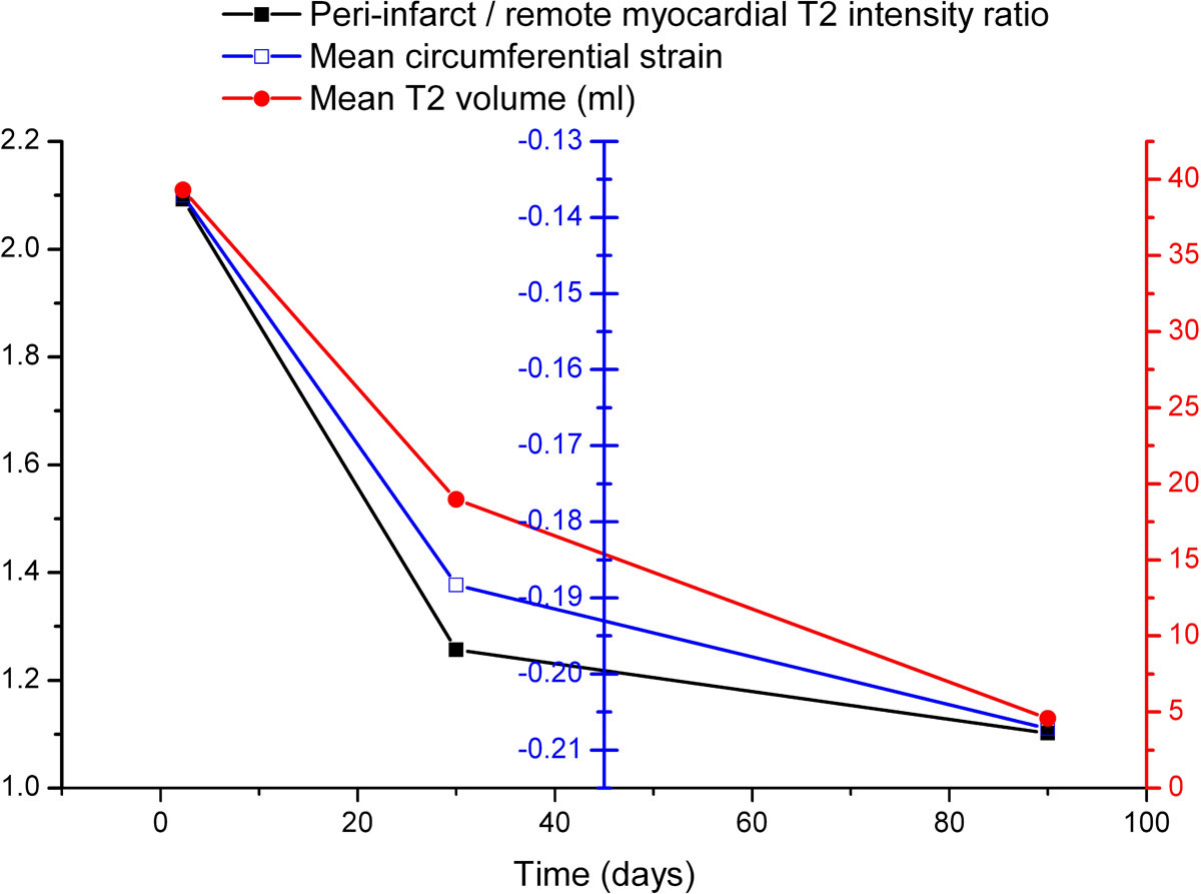
Change in strain, T2 hyperenhancement intensity, and peri-infarct myocardial volume (area at risk) in the peri-infarct zone.

Thirty patients had CMR imaging at all three time points and had adequate image quality with sufficient peri-infarct oedema for quantitative analysis. Circumferential strain was significantly diminished in infarct and peri-infarct zones compared to remote myocardium (means -0.149 vs. -0.184 vs. -0.236, P<0.01 between groups and P<0.01 for trend). Remote myocardium showed no significant change in strain over time (F=1.44, P=0.24), whilst the peri-infarct zone (F=6.03, P=0.004) and infarct zone (F=20.34, P<0.001) showed a significant increase in magnitude. This change closely mirrored resolution of both intensity and volume of T2 hyperenhancement (Figure 1). Decreased circumferential strain correlated significantly with T2 volume (r=0.30; P<0.01) and corrected T2 signal intensity (r=0.28; P<0.01).

Due to the unexpected finding of recovery of function in the infarct zone, we analysed the 8 patients with complete transmural infarction. The area of fully transmural infarction showed a trend towards significant resolution of strain with time (means -0.103 (day 2), -0.148 (day 30) and -0.194 (day 90); P=0.050 for trend).

## Conclusions

In the setting of acute reperfused MI, improvement of strain in stunned myocardium closely follows the regression of myocardial oedema. Patients with larger oedema volumes and higher signal intensity on T2w imaging demonstrated greater improvement of strain within the area at risk. Strain imaging reveals recovery of function within transmurally infarcted myocardium. Volume and intensity of hyperenhancement on T2w CMR in the area at risk may give insights into functional recovery post reperfused MI.

## Funding

S.P is funded by British Heart Foundation fellowship (FS/10/62/28409).

S.P and J.P.G receive a research grant from Philips Healthcare.

